# Blockade of the Arid5a/IL-6/STAT3 axis underlies the anti-inflammatory effect of Rbpjl in acute pancreatitis

**DOI:** 10.1186/s13578-022-00819-1

**Published:** 2022-06-20

**Authors:** Jiachen Lv, Min Fang, Shijie Sun, Gang Wang, Songbin Fu, Bei Sun, Jinxue Tong

**Affiliations:** 1 Second Colorectal Surgery Department, Harbin Medical University Cancer Hospital, No. 150, Haping Road, Nangang District, Harbin, 150001 Heilongjiang People’s Republic of China; 2 Panceatic and Biliary Surgery Department, The First Affiliated Hospital of Harbin Medical University, Harbin, 150001 People’s Republic of China; 3 Genetic Laboratory, Harbin Medical University, Harbin, 150001 People’s Republic of China

**Keywords:** Rbpjl, Arid5a, IL-6, STAT3, Acute pancreatitis, Pancreatic acinar cells, Inflammation

## Abstract

**Background:**

The microarray data analysis predicted that Rbpjl is poorly expressed in acute pancreatitis (AP). Activated IL-6/STAT3 signaling is further known to contribute to the progression of AP through immune regulation, and both IL-6 and STAT3 were bioinformatically predicted to interact with Arid5a. Accordingly, we aimed to investigate the potential involvement of the Arid5a/IL-6/STAT3 axis in the regulatory role of Rbpjl in the inflammation of AP.

**Methods:**

Pancreatic acinar cells were exposed to lipopolysaccharide (LPS) to induce the pancreatic cell damage, and mice were subjected to supramaximal cerulein stimulation to induce AP. Expression patterns of Rbpjl and the Arid5a/IL-6/STAT3 axis were measured in mouse and cell models. Their expression was further manipulated to explore their effects on pancreatic cell injury and inflammation, as reflected by cell viability and apoptosis as well as reactive oxygen species (ROS) accumulation and proinflammatory cytokine secretion. Moreover, ChIP, EMSA, and dual-luciferase reporter assays were carried out to identify the interactions between Rbpjl and Arid5a.

**Results:**

Rbpjl was found to be down-regulated in pancreatic tissues of AP mice and LPS-induced pancreatic acinar cells, while re-expression of Rbpjl led to enhanced cell viability, suppressed LPS-induced inflammation and ROS accumulation, and alleviation of AP-induced damage. Mechanistically, Rbpjl could bind to the promoter region of Arid5a and down-regulated its expression, thus repressing the activation of the IL-6/STAT3 signal axis. Furthermore, Rbpjl impaired Arid5a-dependent IL-6/STAT3 activation, hence alleviating pancreatic acinar cell inflammation. Furthermore, these effects were validated with in vivo experiments.

**Conclusion:**

Collectively, our findings highlight that Rbpjl attenuates AP by down-regulating Arid5a and inactivating the IL-6/STAT3 pathway.

**Supplementary Information:**

The online version contains supplementary material available at 10.1186/s13578-022-00819-1.

## Background

Acute pancreatitis (AP) entails acute inflammation of the pancreas that can precipitate organ failure and secondary pancreatic infection or peripancreatic necrosis, and lead to significant mortality [1, 2]. Pathologically, inflammation of pancreatic acinar cells is regarded a hallmark of AP [3]. Interestingly, characterization of signaling pathways implicated in acinar cell injury, such as inflammatory and autophagic proteins and endoplasmic reticulum stress markers, can aid the identification of potential molecular targets for therapeutic use against AP [4, 5].

Recombination signal binding protein for immunoglobulin kappa J region like (Rbpjl), is associated with a handful of aliases, including RBPL, SUHL and RBPSUHL. Recently, Rbpjl was identified as a protein expressed in pancreas particularly in exocrine pancreas, as well as in endocrine cells that are implicated the development of human pancreas and thus represents a potential target for exploration of pancreas-related disease states and treatment [6, 7]. Moreover, alterations in the pancreas specific Rbpjl in mouse pancreatic acinar cells are known to influence the risk of type 2 diabetes by regulating the expression of exocrine enzyme [6]. Furthermore, replacement of Rbpj with Rbpjl in the pancreas transcription factor 1 (PTF1) complex is associated with acinar differentiation, whereby maintaining the homeostasis in acinar cells [8].

Initial bioinformatics prediction in our study indicated that AT-rich interactive domain-containing protein 5a (Arid5a) serves as a differential gene interacting with Rbpjl. In addition, Arid5a is detected as an RNA-binding protein in both the cytoplasm and nucleus of cells in normal growth, and further exerts a crucial role in the context of inflammation [9]. What’s more, Arid5a also possesses therapeutic potential against inflammatory autoimmune diseases such as encephalomyelitis and sepsis [10]. Existing evidence further indicates that Arid5a is capable of stabilizing interleukin 6 (IL-6), signal transducers and activators of transcription 3 (STAT3) to exert its effects of immune regulation [11]. IL-6 represents a member of prototypical cytokines that participate in the maintenance of homeostasis [12]. Meanwhile, STAT3, a cellular signal transcription factor, is known to regulate a variety of cellular activities, including cell differentiation, proliferation, and angiogenesis in normal cells [13]. Furthermore, activation of the IL-6/STAT3 axis is associated with aggravation of AP [14], whereas down-regulation of this axis by microRNA-148a brought about suppression of autophagy in AP induced by cerulein [15].

In lieu of the aforementioned evidence, we proposed a hypothesis that Rbpjl might affect AP through its regulation on Arid5a and the IL-6/STAT3 axis. Accordingly, the current study set out to testify the aforementioned hypothesis and illuminate the underlying molecular mechanism of Rbpjl in pancreatic acinar cell and animal models of AP, in an effort to provide novel therapeutic strategies against AP.

## Results

### Rbpjl was poorly expressed in pancreatic tissues of AP mice and lipopolysaccharide (LPS)-induced pancreatic acinar cells

**Fig. 1:**
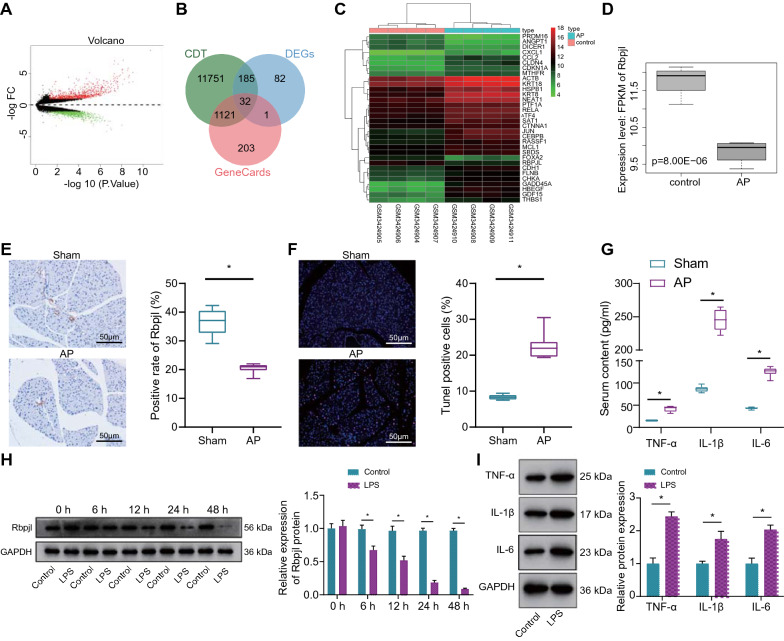
Rbpjl is under-expressed in both pancreatic tissues of AP mice and LPS-induced pancreatic acinar cells. **A** A volcano map of the expression of DEGs in AP samples in GSE121038 microarray (red dot indicates highly expressed genes, green dot indicates poorly expressed genes, X axis represents -log10, and Y axis indicates logFC. **B** Venn map of AP-related genes from GeneCards and CDT databases and differential genes obtained from GEO database. **C** A heat map of 32 candidate genes involved in the regulation of AP in AP samples (green to red indicates the expression value from small to large). **D** The expression of Rbpjl in AP samples in the GSE121038 microarray. **E** Immunohistochemistry analysis of Rbpjl protein in pancreatic tissues of sham-operated or AP mice (* *p* < 0.05 *vs.* sham-operated mice). **F** The apoptosis rate in pancreatic tissues of sham-operated or AP mice was detected by TUNEL staining (* *p* < 0.05 *vs.* sham-operated mice). **G** The expression of pro-inflammatory factors in the serum of sham-operated or AP mice was detected by ELISA (* *p* < 0.05 *vs.* sham-operated mice). H, Western blot assay was used to detect the protein expression of Rbpjl in control pancreatic acinar cells or pancreatic acinar cells treated with LPS at different time points (* *p* < 0.05 *vs.* control cells). **I** The expression of pro-inflammatory factors in control pancreatic acinar cells or pancreatic acinar cells treated with LPS for 24 h was detected by Western blot assay. (* *p* < 0.05 *vs.* control cells). The mouse experiments were performed 14 days after AP modeling, with n = 8 for mice in each group. The cell experiment was conducted three times independently

First, we retrieved the AP-related gene expression microarray dataset GSE121038 (Additional file 1: Table S1) from the GEO database. Next, differentially expressed genes (DEGs) were screened in the GSE121038 dataset, which yielded a total of 1549 DEGs, including 826 up-regulated genes and 723 down-regulated genes (Fig. 1A). Additionally, 1357 and 13,089 AP-related genes were obtained from the GeneCards and CDT databases, respectively. Subsequently, 32 genes were obtained by taking the intersection of the top 300 DEGs in the GSE121038 dataset and the AP-related genes (Fig. 1B). The detailed differential analysis results of the 32 genes are illustrated in Additional file 2: Table S2. In accordance with the expression data of the 32 genes in control and AP samples of the GSE121038 dataset in Additional file 3: Table S3, an expression heat map of these 32 genes was plotted, which revealed that Foxa2, PRDM16, ANGPT1, Dicer1 and Rbpjl were significantly down-regulated in AP (Fig. 1C). Rbpjl is regarded as a key gene for the differentiation and maturation of acinar cells, and further implicated in maintaining the homeostasis of acinar cells to drive acinar differentiation [8, 16, 17]. Consistently, results of microarray GSE121038 profiling displayed that Rbpjl was poorly expressed in AP samples (Fig. 1D), suggesting that Rbpjl may participate in the occurrence of AP, and thus was selected as the focus of our study.

Furthermore, results of hematoxylin and eosin (HE) staining illustrated that compared with sham-operated mice, the pancreatic tissue integrity in AP mice was completely destroyed, accompanied by severe morphological changes of interstitial edema, interstitial hemorrhage and fat necrosis, as well as severe structural damage (Additional file 5: Figure S1A). Meanwhile, pancreatic organ coefficient in AP mice was significantly increased relative to sham-operated mice (Additional file 5: Fig. S1SB). These findings highlighted the successful establishment of AP mouse models.

Immunohistochemical staining further demonstrated that the expression of Rbpjl was significantly lower in pancreatic tissues of AP mice relative to that in sham-operated mice (Fig. 1E). Moreover, the results of terminal deoxynucleotidyl transferase-mediated dUTP-biotin nick end labeling (TUNEL) staining illustrated that the apoptosis rate of pancreatic cells was notably higher in AP mice compared to that in sham-operated mice (Fig. 1F). Subsequent enzyme-linked immunosorbent assay (ELISA) results revealed that compared with sham-operated mice, the expression of pro-inflammatory factors TNF-α, IL-1β and IL-6 was all significantly increased in the serum of AP mice (Fig. 1G).

Additionally, the results of Western blot assay displayed that pancreatitis-associated protein-I (PAP-I) protein expression [18] was almost undetectable in control pancreatic acinar cells, while being highly expressed in LPS-induced MPC-83 cells (Additional file 5: Fig. S1C). ELISA results also depicted that the expression of TNF-α, IL-1β and IL-6 was significantly elevated in the supernatant of LPS-induced MPC-83 cells (Additional file 5: Fig. S1D). These findings highlighted the successful establishment of in vitro pancreatic cell injury model.

Moreover, Western blot assay results illustrated that expression of Rbpjl was progressively reduced in LPS-induced MPC-83 cells over time than that in control MPC-83 cells, whereas, the reduction was no longer obvious after 24 h (Fig. 1H). In addition, there was an increase in the expression of TNF-α, IL-1β and IL-6 in the supernatant of LPS-induced MPC-83 cells (Fig. 1I).

Altogether, the aforementioned findings demonstrated that Rbpjl was poorly expressed in both mouse AP models and pancreatic cell injury models.

### Rbpjl inhibited LPS-induced inflammatory response in pancreatic acinar cells

**Fig. 2:**
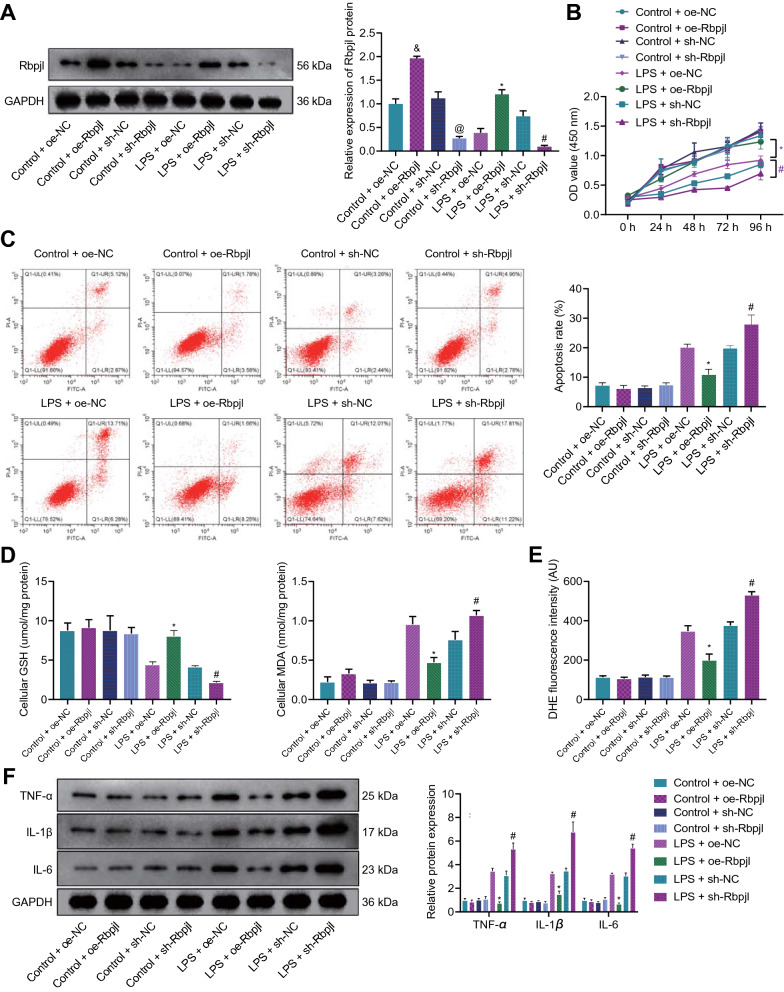
Overexpression of Rbpjl inhibits LPS-induced inflammatory response in pancreatic acinar cells. Control and LPS-induced MPC-83 cells were treated with oe-Rbpjl or sh-Rbpjl. **A** The protein expression of Rbpjl in control and LPS-induced MPC-83 cells was detected by Western blot assay. **B** The control and LPS-induced MPC-83 cell viability was detected by CCK-8 assay. **C** Apoptosis of control and LPS-induced MPC-83 cells detected by flow cytometry. **D** GSH and MDA production in control and LPS-induced MPC-83 cells were detected using an ultraviolet spectrophotometer. **E** The production of ROS in control and LPS-induced MPC-83 cells. **F** The expression of pro-inflammatory factors in control and LPS-induced MPC-83 cells was detected by Western blot assay. & *p* < 0.05 *vs.* control MPC-83 cells + oe-NC. @ *p* < 0.05 *vs.* control MPC-83 cells + sh-NC. * *p* < 0.05 *vs.* MPC-83 cells treated with LPS + oe-NC. # *p* < 0.05 *vs.* MPC-83 cells treated with LPS + sh-NC. Cell experiments were performed after 72 h of lentivirus transduction and 24 h of LPS stimulation. The cell experiment was conducted three times independently

To further elucidate the effect of Rbpjl on AP, we over-expressed or knocked down Rbpjl in the LPS-induced MPC-83 cells and determined the efficiency of oe-Rbpjl/sh-Rbpjl. As illustrated by the results of Western blot assay, oe-Rbpjl treatment in control MPC-83 cells elevated Rbpjl expression, while sh-Rbpjl treatment brought about the opposing effects. In addition, oe-Rbpjl treatment in LPS-exposed MPC-83 cells led to elevated Rbpjl expression and sh-Rbpjl treatment resulted in a reduction of Rbpjl expression. Additionally, LPS-induced MPC-83 cells following each treatment presented with lower Rbpjl expression compared to control MPC-83 cells following each treatment (Fig. 2A).

Moreover, over-expression of Rbpjl led to enhanced LPS-induced MPC-83 cell proliferation, while these effects were abrogated by Rbpjl silencing. Meanwhile, there were no evident differences in the proliferation of each group of MPC-83 cells without LPS stimulation (Fig. 2B). Following LPS treatment, MPC-83 cell apoptosis was reduced following over-expression of Rbpjl. Conversely, Rbpjl silencing resulted in enhanced cell apoptosis. Each group of MPC-83 cells without LPS stimulation exhibited no significant difference in the apoptosis, whereas apoptosis of LPS-induced MPC-83 cells was higher following each treatment as compared to control cells (Fig. 2C).

Over-expression of Rbpjl increased glutathione (GSH) production, while down-regulating that of malondialdehyde (MDA) in LPS-induced MPC-83 cells. Conversely, GSH production was decreased, and MDA production was increased following Rbpjl silencing. There were no changes in GSH and MDA production in the control MPC-83 cells following each treatment, while LPS-induced MPC-83 cells upon each treatment presented with lower GSH production and higher MDA production relative to MPC-83 cells without LPS stimulation (Fig. 2D). Moreover, over-expression of Rbpjl notably reduced reactive oxygen species (ROS) accumulation, whereas Rbpjl silencing led to increased ROS accumulation. Meanwhile, LPS-induced MPC-83 cells following each treatment exhibited higher ROS accumulation compared to MPC-83 cells without LPS stimulation (Fig. 2E). Furthermore, the results of Western blot assay and ELISA illustrated that over-expression of Rbpjl decreased the levels of pro-inflammatory proteins TNF-α, IL-1β and IL-6 in LPS-induced MPC-83 cells and the supernatant, respectively, while opposite results were observed following Rbpjl knockdown. In addition, the levels of TNF-α, IL-1β and IL-6 were not significantly altered in each group of MPC-83 cells without LPS stimulation, while LPS-induced MPC-83 cells following each treatment presented with higher levels relative to MPC-83 cells without LPS stimulation (Fig. 2F, Additional file 5: Figure S1E).

Overall, the aforementioned findings suggested that over-expression of Rbpjl attenuated the damage of LPS to pancreatic cells and reduced the inflammatory response.

### Rbpjl targeted and inversely regulated Arid5a

**Fig. 3:**
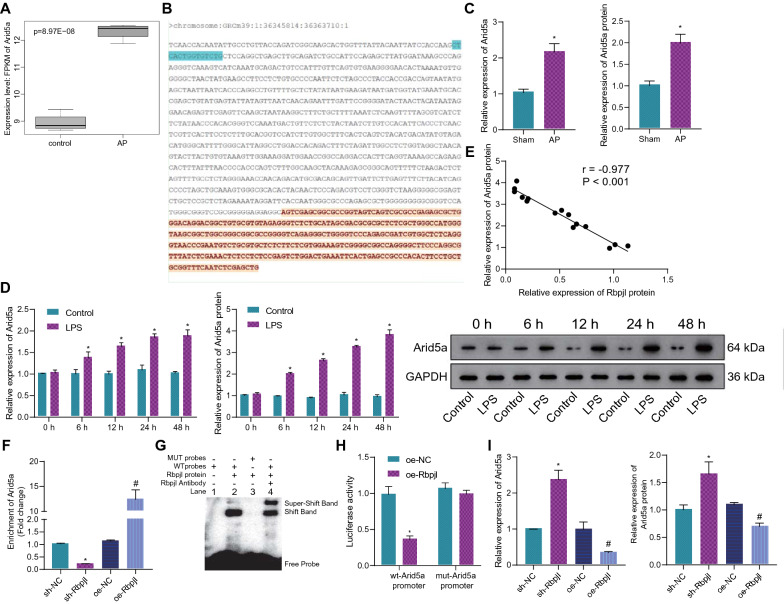
Rbpjl down-regulates the expression of Arid5a by binding to its promoter region. **A** Analysis of the expression of Arid5a in AP samples in the GSE121038 dataset. **B** The binding site of Rbpjl and the Arid5a promoter region was predicted by the JASPAR website. **C** Detection of mRNA and protein expression of Arid5a in the pancreatic tissues of AP mice by RT-qPCR and Western blot assay (* *p* < 0.05 *vs.* sham-operated mice). **D** Detection of mRNA and protein expression of Arid5a in the LPS-induced MPC-83 cells at different time points by RT-qPCR and Western blot assay (* *p* < 0.05 *vs.* control cells). **E** Correlation of Arid5a protein expression with Rbpjl protein expression in LPS-induced MPC-83 cells at different time points analyzed by Pearson's correlation coefficient (* *p* < 0.0001). **F** The binding of Rbpjl to the Arid5a promoter region was analyzed by ChIP after pancreatic acinar cells with oe-Rbpjl or sh-Rbpjl (for 72 h) were stimulated by LPS (for 24 h) (* *p* < 0.05 *vs.* cells treated with sh-NC. ^#^ *p* < 0.05 *vs.* cells treated with oe-NC). G, Rbpjl protein binding to the Arid5a promoter region in pancreatic acinar cells was analyzed by EMSA. **H** Luciferase activity of WT-Arid5a promoter and MUT-Arid5a promoter in HEK-293 T cells transfected with oe-Rbpjl was detected by dual luciferase reporter assay (* *p* < 0.05 *vs.* HEK-293 T cells transfected with oe-NC). **I** The mRNA and protein expression of Arid5a was measured by RT-qPCR and Western blot assay after pancreatic acinar cells with oe-Rbpjl or sh-Rbpjl (for 72 h) were stimulated by LPS (for 24 h) (* *p* < 0.05 *vs.* cells treated with sh-NC. ^#^ *p* < 0.05 *vs.* cells treated with oe-NC). The mouse experiments were performed 14 days after AP modeling, with n = 8 for mice in each group. The cell experiment was conducted three times independently

Next, we sought to further investigate the molecular mechanism of Rbpjl in AP. There is evidence to suggest that Arid5a is highly expressed in the context of inflammation [19]. In this study, Arid5a expression was up-regulated in AP samples in the GSE121038 dataset (Fig. 3A). Moreover, the JASPAR website predicted that the binding sequence of transcription factor Rbpjl and gene Arid5a promoter region was CAGACACCAGTGAG (Fig. 3B). Subsequent results of reverse transcription-quantitative polymerase chain reaction (RT-qPCR) and Western blot assay confirmed that Arid5a was indeed highly expressed in the pancreatic tissues of AP mice (Fig. 3C, Additional file 6: Figure S2A). Meanwhile, the expression of Arid5a showed a progressive increase in the LPS-induced MPC-83 cells over time, while this increase was no longer prominent after 24 h (Fig. 3D). In addition, Pearson's correlation coefficient illustrated that Arid5a protein expression was negatively correlated with Rbpjl protein expression in LPS-induced MPC-83 cells at different time points (Fig. 3E). Therefore, we speculated that Rbpjl might affect AP by regulating the expression of Arid5a.

Accordingly, we manipulated the expression of Rbpjl in pancreatic acinar cells, with over-expression and knockdown efficiency validated by RT-qPCR and Western blot assay (Additional file 7: Figure S3A). The results of chromatin immunoprecipitation (ChIP) assay revealed that enrichment of Arid5a promoter by Rbpjl was reduced in the presence of Rbpjl silencing, while being increased following Rbpjl over-expression (Fig. 3F), underscoring that Rbpjl could bind to the Arid5a promoter region. Moreover, the results of electrophoresis mobility shift assay (EMSA) further revealed that Rbpjl bound to the Arid5a promoter-WT, but not to the Arid5a promoter-MUT (Fig. 3G). Meanwhile, dual luciferase reporter assay data identified a significant decrease in the luciferase activity of WT-Arid5a promoter following Rbpjl overexpression, whereas there were no alterations in the luciferase activity of MUT-Arid5a promoter (Fig. 3H).

Furthermore, the results of RT-qPCR and Western blot assay illustrated that knockdown of Rbpjl led to a significant enhancement in the mRNA and protein expression of Arid5a, while over-expression of Rbpjl led to the opposite results (Fig. 3I, Additional file 6: Figure S2B).

Altogether, the aforementioned findings suggested that Rbpjl specifically bound to Arid5a promoter and negatively regulated the expression of Arid5a.

### Arid5a activated the IL-6/STAT3 axis in pancreatic acinar cells

**Fig. 4:**
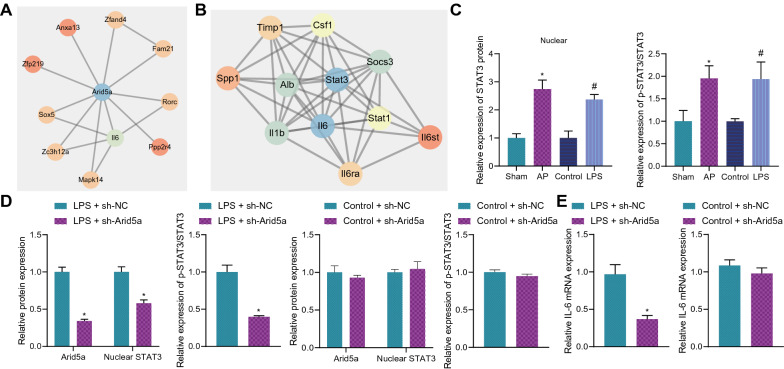
Arid5a activates the IL-6/STAT3 axis in pancreatic acinar cells. **A** The interaction network of Arid5a in the STRING website. **B** The interaction network of IL-6 in the STRING website. **C** Detection of intranuclear protein expression of STAT3 and STAT3 phosphorylation level in the pancreatic tissues of AP mice and LPS-induced MPC-83 cells (for 6 h) by Western blot assay (* *p* < 0.05 *vs.* sham-operated mice. ^#^ *p* < 0.05 *vs.* control MPC-83 cells). **D** The expression of intranuclear STAT3 protein, Arid5a, total protein expression of STAT3 as well as STAT3 phosphorylation level in the LPS-induced MPC-83 cells (for 6 h) treated with sh-Arid5a was measured by Western blot assay (* *p* < 0.05 *vs.* LPS-induced MPC-83 cells treated with sh-NC). **E** The expression of pro-inflammatory factor IL-6 in LPS-induced MPC-83 cells (for 6 h) treated with sh-Arid5a was detected by RT-qPCR (* *p* < 0.05 *vs.* LPS-induced MPC-83 cells treated with sh-NC). The mouse experiments were performed 14 days after AP modeling, with n = 8 for mice in each group. The cell experiment was conducted three times independently

To further predict the downstream regulatory factors of Arid5a, the STRING website was utilized to obtain the gene interaction network of Arid5a. Subsequent findings revealed that IL-6 was located at the core of the network, exhibiting a close interaction with Arid5a (Fig. 4A), whereas STAT3 was located as the core of IL-6 interaction network, exhibiting close association with IL-6 (Fig. 4B). As illustrated by Western blot assay results, the intranuclear protein expression of STAT3 and STAT3 phosphorylation levels were significantly increased, while the total protein expression of STAT3 did not alter in the pancreatic tissues of AP mice and LPS-induced MPC-83 cells (Fig. 4C, Additional file 6: Figure S2C). Following knockdown of Arid5a in LPS-induced MPC-83 cells, there was a decrease in the intranuclear protein expression of STAT3 and STAT3 phosphorylation levels, whereas the total protein expression of STAT3 was unaffected. In addition, in MPC-83 cells without LPS stimulation, knockdown of Arid5a did not alter the total protein expression of STAT3 (Fig. 4D, Additional file 6: Figure S2D). Moreover, the results of RT-qPCR exhibited that the IL-6 levels were decreased in LPS-induced MPC-83 cells in response to Arid5a knockdown. Meanwhile, there were no significant changes in the level of IL-6 in the MPC-83 cells upon Arid5a knockdown without LPS stimulation (Fig. 4E).

Collectively, the aforementioned findings indicated that Arid5a activated the IL-6/STAT3 axis in pancreatic acinar cells.

### Rbpjl impaired Arid5a-dependent activation of IL-6/STAT3 axis to alleviate inflammatory response

**Fig. 5:**
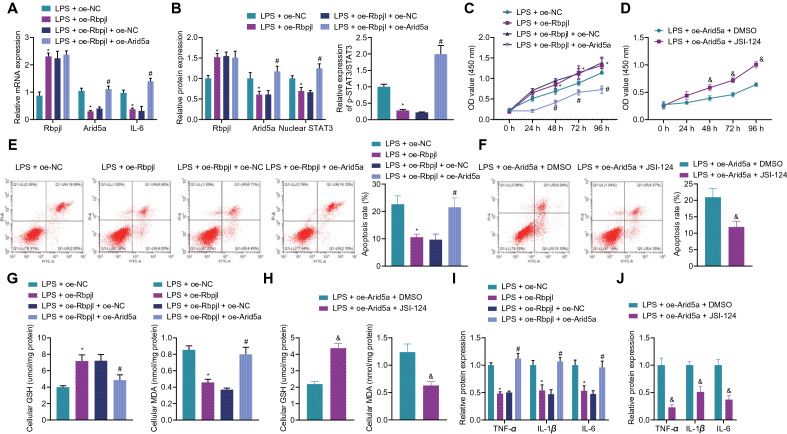
Rbpjl downregulates Arid5a expression and thus inhibits the IL-6/STAT3 axis to alleviate inflammatory response in LPS-induced pancreatic acinar cells. **A** mRNA expression of Rbpjl, Arid5a and IL-6 after MPC-83 cells with oe-Rbpjl or combined with oe-Arid5a (for 72 h) were stimulated by LPS (for 24 h) determined by RT-qPCR. **B** Western blot assay was used to detect the expression of Rbpjl, Arid5a and intranuclear STAT3 proteins, total protein expression of STAT3 as well as STAT3 phosphorylation level after MPC-83 cells with oe-Rbpjl or combined with oe-Arid5a (for 72 h) were stimulated by LPS (for 24 h). **C** CCK-8 assay was used to detect the proliferation of MPC-83 cells with oe-Rbpjl or combined with oe-Arid5a (for 72 h) stimulated by LPS (for 24 h). **D** CCK-8 assay was used to detect the proliferation of MPC-83 cells with oe-Arid5a (for 72 h) or combined with JSI-124 (for 24 h) stimulated by LPS (for 24 h). **E** Apoptosis of MPC-83 cells with oe-Rbpjl or combined with oe-Arid5a (for 72 h) stimulated by LPS (for 24 h) detected by flow cytometry. **F** Apoptosis of MPC-83 cells with oe-Arid5a (for 72 h) or combined with JSI-124 (for 24 h) stimulated by LPS (for 24 h) detected by flow cytometry. **G** Ultraviolet spectrophotometers were used to detect GSH and MDA production in MPC-83 cells with oe-Rbpjl or combined with oe-Arid5a (for 72 h) stimulated by LPS (for 24 h). **H** Ultraviolet spectrophotometers were used to detect GSH and MDA production in MPC-83 cells with oe-Arid5a (for 72 h) or combined with JSI-124 (for 24 h) stimulated by LPS (for 24 h). **I** TNF-α, IL-1β and IL-6 expression in MPC-83 cells with oe-Rbpjl or combined with oe-Arid5a (for 72 h) stimulated by LPS (for 24 h). **J** TNF-α, IL-1β and IL-6 expression in MPC-83 cells with oe-Arid5a (for 72 h) or combined with JSI-124 (for 24 h) stimulated by LPS (for 24 h). * *p* < 0.05 *vs.* MPC-83 cells treated with LPS + oe-NC. ^#^ *p* < 0.05 *vs.* MPC-83 cells treated with LPS + oe-Rbpjl + oe-NC. & *p* < 0.05 *vs.* MPC-83 cells treated with LPS + oe-Arid5a + DMSO. The cell experiment was conducted three times independently

In an attempt to further explore the effect of Rbpjl regulating the Arid5a/IL-6/STAT3 axis on inflammatory response of pancreatic acinar cells, we manipulated the expression of Rbpjl and Arid5a in pancreatic acinar cells. Subsequent results of RT-qPCR illustrated an enhancement of Rbpjl mRNA expression and a decline in Arid5a and IL-6 mRNA expression in LPS-induced MPC-83 cells transfected with oe-Rbpjl. Meanwhile, there were no changes in the Rbpjl mRNA expression, while Arid5a and IL-6 mRNA expression was up-regulated following further over-expression of Arid5a. However, in each group of MPC-83 cells without LPS stimulation, Arid5a and IL-6 mRNA expression exhibited consistent results to those of LPS-induced MPC-83 cells following each treatment, except for the unaltered IL-6 mRNA expression in the MPC-83 cells without LPS stimulation (Fig. 5A, Additional file 8: Figure S4A). Moreover, Western blot assay results demonstrated that over-expression of Rbpjl decreased the intranuclear protein expression of STAT3 and STAT3 phosphorylation levels, while the total protein expression of STAT3 was unaffected in LPS-induced MPC-83 cells, and these effects could be reversed by restoration of Arid5a. Each group of MPC-83 cells without LPS stimulation exhibited consistent results in the protein expression of Rbpjl and Arid5a as those of LPS-induced MPC-83 cells following each treatment; there were no appreciable differences in the intranuclear protein expression of STAT3, total protein expression of STAT3, or STAT3 phosphorylation levels in each group of MPC-83 cells without LPS stimulation (Fig. 5B, Additional file 6: Figure S2E and Additional file 8: Figure S4B).

Furthermore, cell counting kit-8 (CCK-8) assay results illustrated that over-expression of Rbpjl promoted the viability of LPS-induced MPC-83 cells, while these effects were reversed by over-expression of Arid5a. In addition, following treatment with oe-Arid5a + JSI-124 (STAT3 inhibitor, 1 µM for 24 h, Bio-Techne Co. Ltd.), LPS-induced MPC-83 cells presented with enhanced cell viability. Meanwhile, there were no changes in cell viability in each group of MPC-83 cells without LPS stimulation (Fig. 5C, D, Additional file 8: Figure S4C, D). The results of flow cytometry showed that over-expression of Rbpjl inhibited MPC-83 cell apoptosis, whereas over-expression of Arid5a promoted cell apoptosis. On the other hand, JSI-124 treatment reversed the promoting effect of Arid5a over-expression on the cell apoptosis. Each group of MPC-83 cells without LPS stimulation exhibited no differences in the cell apoptosis (Fig. 5E, F, Additional file 8: Figure S4E, F).

Moreover, over-expression of Rbpjl significantly increased the production of GSH and down-regulated the production of MDA in LPS-induced MPC-83 cells, whereas over-expression of Arid5a reversed the effects of Rbpjl on the production of GSH and MDA. In addition, following treatment with oe-Arid5a + JSI-124, the production of GSH was elevated and that of MDA was reduced in the LPS-induced MPC-83 cells. Meanwhile, there were no changes in the production of GSH and MDA in each group of MPC-83 cells without LPS stimulation (Fig. 5G, H, Additional file 8: Figure S4G, H). Additionally, the results of ELISA and Western blot assay demonstrated that Rbpjl over-expression significantly inhibited the secretion of inflammatory factors in LPS-induced MPC-83 cells and the supernatant, respectively, while this anti-inflammatory effect was negated by overexpression of Arid5a. On the other hand, JSI-124 treatment reversed the promotive effect of Arid5a over-expression on the secretion of inflammatory factors. Besides, secretion of inflammatory factors showed no alterations in each group of MPC-83 cells without LPS stimulation (Fig. 5I, J, Additional file 6: Figure S2F, G, Additional file 7: Figure S3B, C, Additional file 8: Figure S4I, J).

Overall, the aforementioned findings suggested that Rbpjl decreased the expression of Arid5a and thus inhibited IL-6/STAT3 axis to alleviate LPS-induced inflammatory response in pancreatic acinar cells.

### Rbpjl alleviated AP by blocking Arid5a-dependent activation of IL-6/STAT3 in vivo

**Fig. 6:**
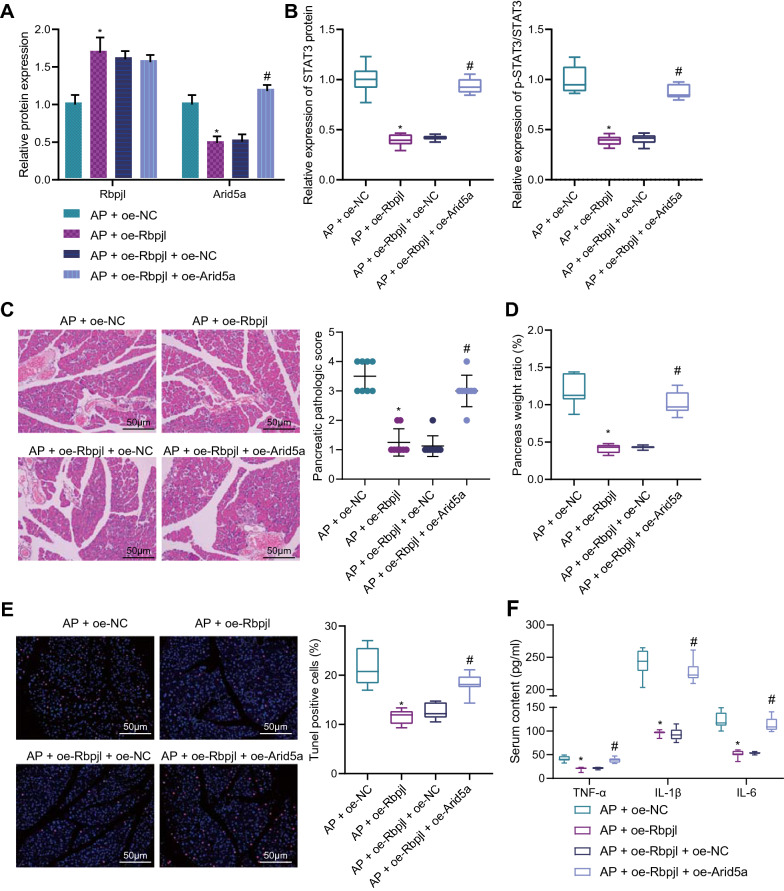
Rbpjl alleviates AP by downregulating Arid5a and blocking the IL-6/STAT3 axis in vivo. **A** Rbpjl and Arid5a protein expression in pancreatic tissues of mice treated with oe-Rbpjl or combined with oe-Arid5a determined by Western blot assay. **B** The intranuclear STAT3 protein expression, total protein expression of STAT3 as well as STAT3 phosphorylation level in pancreatic tissues of mice treated with oe-Rbpjl or combined with oe-Arid5a were measured by Western blot assay. **C** HE staining was used to observe the degree of pancreatic injury, and the degree of pancreatic injury was graded and evaluated in mice treated with oe-Rbpjl or combined with oe-Arid5a. **D** The weight ratio of pancreatic tissues to total body in mice treated with oe-Rbpjl or combined with oe-Arid5a was detected. **E** TUNEL staining was used to detect the apoptosis rate in pancreatic tissues of mice treated with oe-Rbpjl or combined with oe-Arid5a. **F** The expression of pro-inflammatory factors in the serum of mice treated with oe-Rbpjl or combined with oe-Arid5a was detected by ELISA. The mouse experiments were performed 14 days after AP modeling, with n = 8 for mice in each group

Lastly, we established mouse models of AP, and then injected lentivirus over-expressing Rbpjl or Arid5a into mice via tail vein. Subsequent results of Western blot assay illustrated that over-expression of Rbpjl augmented the protein expression of Rbpjl and reduced that of Arid5a in pancreatic tissues of AP mice. However, there were no changes in Rbpjl protein expression, while the Arid5a protein expression was elevated in pancreatic tissues of oe-Rbpjl + oe-Arid5a-treated mice (Fig. 6A, Additional file 6: Figure S2H). Moreover, Western blot assay results showed that over-expression of Rbpjl notably decreased intranuclear STAT3 protein expression and STAT3 phosphorylation levels, while the total protein expression of STAT3 remained unaltered in pancreatic tissues of AP mice; however, further over-expression of Arid5a reversed the effects of Rbpj1 over-expression (Fig. 6B, Additional file 6: Figure S2I).

Furthermore, HE staining results illustrated that over-expression of Rbpjl significantly alleviated pancreatic tissue lesions in AP mice; whereas, over-expression of Arid5a led to significant aggravation of severe pancreatic tissue lesions in the presence of Rbpjl (Fig. 6C). Moreover, over-expression of Rbpjl markedly decreased pancreatic organ coefficient in AP mice, while these effects were reversed by over-expression of Arid5a (Fig. 6D). Moreover, the results of TUNEL staining depicted that over-expression of Rbpjl significantly reduced cell apoptosis in pancreatic tissues of AP mice, which could be reversed by over-expression of Arid5a (Fig. 6E). In addition, ELISA results displayed that over-expression of Rbpjl significantly inhibited the expression of pro-inflammatory factors in the serum of AP mice, while these effects could be abrogated by over-expression of Arid5a (Fig. 6F).

Collectively, the aforementioned findings indicated that Rbpjl was capable of alleviating AP by down-regulating Arid5a and inhibiting the IL-6/STAT3 axis in vivo.

## Discussion

In our current work, we validated the alleviatory effect of Rbpjl in AP through a series of functional experiments both in vitro and in vivo, in an effort to offer potential future therapeutic strategies against AP. Altogether, our findings highlighted that Rbpjl alleviated the development of AP by regulating the Arid5a/IL-6/STAT3 axis.

The obtained data illustrated that Rbpjl was poorly expressed in the pancreatic tissues of AP mice and LPS-induced pancreatic acinar cells, whereas further over-expression of Rbpjl exerted an inhibitory effect on LPS-induced inflammatory response in pancreatic acinar cells. Unsurprisingly, Rbpjl was previously indicated to serve as an important transcription factor in the regulation of the differentiation and development of acinar cells [16]. Further elaborating the significance of Rbpj1 in pancreatic disorders, the study carried out by Nair et al. indicated that Rbpjl could affect type 2 diabetes risk via positive modulation of exocrine enzyme Ctrb in mouse pancreatic acinar cells [6]. Meanwhile, PTF1a, functioning as a key transcription factor regulating early pancreatic development, is capable of directly binding to the promoter region of Rbpjl gene, while also being implicated in the transcription initiation of Rbpjl [20]. Recent investigations have further shown that PTF1a is poorly expressed at the onset of pancreatitis [21], while one study remarked that deletion of PTF1a alone is enough to precipitate inflammatory response of pancreatic cells [22]. Altogether, the aforementioned findings and evidence make it plausible to suggest that down-regulation of Rbpjl expression may be directly associated with down-regulation of PTF1a transcriptional activity in the occurrence of AP, while the regulation of these transcription factors by inflammatory signals requires further exploration.

Additional experimentation in our study identified Arid5a as the downstream factor of Rbpjl in AP through database-based bioinformatics prediction, as well as ChIP and Western blot assays. Currently, Arid5a is regarded as an RNA binding protein that stabilizes various inflammatory factors, such as IL-6 and STAT3 [10]. Interestingly, the loss of Arid5a in mouse models was correlated with resistance to bleomycin-induced lung damage, which is a leading cause of mortality on a global scale owing to acute inflammation in lung [23]. Moreover, a prior study indicated that down-regulation of Arid5a led to diminished levels of IL-6 in LPS-treated mice, and further prevented development of experimental autoimmune encephalomyelitis [24]. Consistent with these findings, the results obtained in this study indicated that the inhibitory role of Rbpjl in AP required downregulation of Arid5a.

Thereafter, we adopted a mechanistic approach in our research, and uncovered that down-regulation of Arid5a inhibited the IL-6/STAT3 axis to ameliorate AP. Strikingly, there is a plethora of evidence highlighting the regulatory relationship between Arid5a and IL-6/STAT3. For instance, Arid5a, functioning as a type of dynamic molecule, was previously shown to stabilize the mRNA transcripts of multiple inflammatory factors, including IL-6, thereby participating in the inflammatory response and progression of inflammation-related diseases [19]. In addition, a prior study demonstrated that targeting the phosphorylation and degradation of Arid5a could facilitate the release of IL-6, consequently exerting a critical role in autoimmunity [25]. Similarly, the efforts of Masuda et al. revealed that Arid5a possesses the ability to promote cytokine production and control the half-life of pro-inflammatory factors, including IL-6 and STAT3, in activated macrophages, as well as T cells [26]. On the other hand, up-regulation of Arid5a induced by β2-adrenergic stimulation was previously associated with elevated IL-6 levels, thereby enhancing inflammation in cardiac fibroblasts [27]. Meanwhile, Arid5a-induced selective stabilization of STAT3 is known to influence the fate of CD4 + T cells [28]. In fact, a large number of studies have explored the critical involvement of IL-6 and STAT3 in AP. For example, inhibition of IL-6 and down-regulation of STAT3 phosphorylation by Chaiqin Chengqi decoction can lead to amelioration of AP [29]. Besides, Wang et al*.* reported that IL-6 up-regulates the expression of TMEM16A through activation of the IL-6R/STAT3 signaling pathway in pancreatic acinar cells, thereby contributing to the development of AP [30]. In contrast, inhibition of IL-6 by picroside II aids the attenuation of intestinal barrier injury induced by severe AP [31]. Overall, these findings underscored the critical involvement of Arid5a-mediated activation of the IL-6/STAT3 axis in the pathogenesis of AP, whereas Rbpjl impaired this activation to attenuate the pancreatic damage, further evidenced by our animal experimentation findings.

In addition to Rbpjl, the Foxa2, PRDM16, ANGPT1 and Dicer1 genes were all uncovered to be significantly down-regulated in AP samples. Foxa2 is regarded as an indicator of the differentiation of islet pancreatic cells, whereas induction of islet pancreatic cell differentiation may be one of the mechanisms of anti-inflammatory effect [32]. Meanwhile, the study performed by Sugiyama et al*.* revealed that PRDM16 is required for pancreatic islet development in vivo [33]. In addition, ANGPT1 gene-modified human mesenchymal stem cells were previously indicated as a potential therapeutic approach for severe AP in rats, due to its ability to diminish pancreatic injury and serum levels of pro-inflammatory cytokines [34]. Lastly, Dicer1 is known to play key roles in the morphogenesis of developing tissues, which is imperative to the maintenance of adult pancreas [35]. In lieu of the aforementioned reports, we cannot exclude the involvement of the above factors in the regulation of AP progression. Moreover, a variety of inflammatory molecular pathways are implicated in the development of pancreatitis, such as NF-κB, MAPK and JAK-STAT [36]. At this conjecture, our study primarily identified the correlation between the inflammatory response mediated by IL-6/STAT3 and the regulation of Rbpjl/Arid5a; however, it cannot be ruled out whether other inflammatory phenotypes are dependent on the Arid5a/IL-6/STAT3 axis, and shall be one of the focuses of our future endeavors.

## Conclusions

**Fig. 7:**
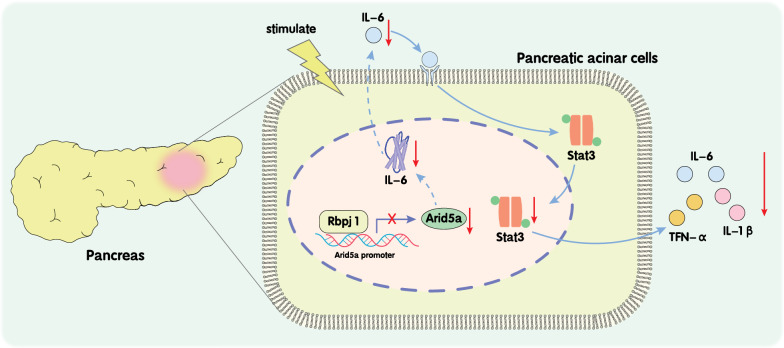
The molecular mechanism plot for the role of Rbpjl in AP. Rbpjl down-regulates Arid5a expression by binding to the Arid5a promoter region, thus reducing IL-6 expression and STAT3 protein phosphorylation, and STAT3 translocation into nuclei. Further, Rbpjl inhibits activation of the IL-6/STAT3 signaling pathway, and the production of inflammatory factors TNF-α, IL-1β and IL-6, thereby alleviating AP

Collectively, findings obtained in our study highlighted that Rbpjl decreases Arid5a expression by binding to the Arid5a promoter region, and blocks activation of the IL-6/STAT3 signaling pathway, thus suppressing the expression of inflammatory factors TNF-α, IL-1β and IL-6 in AP. On the other hand, augmenting the expression of Rbpjl reduces the inflammatory response mediated by Arid5a/IL-6/STAT3, and then attenuates the progression of AP (Fig. 7). Our discoveries could pave the way for promising molecule targets for treatment of AP but requires further confirmation with clinical trials. In addition, the inducing factors and underlying functioning of Rbpjl as an anti-inflammatory mediator during AP warrant investigation in future studies. Lastly, the mechanism behind the regulation of the IL-6/STAT3 signaling by Arid5a in AP warrants further exploration.

## Methods

### Ethical approval

The current study was carried out with the approval of the Ethics Committee of Harbin Medical University Cancer Hospital, and all animal experimental procedures were performed in accordance with the Guide for the Care and Use of Laboratory Animals published by the US National Institutes of Health. Extensive efforts were made to minimize the suffering of the included animals.

### Microarray-based gene expression profiling

The AP-related microarray GSE121038, with better data availability and quality obtained from 16 microarrays, was retrieved from the GEO database with “acute pancreatitis” serving as the key word, “Mus musculus” as the species and “Expression profiling by array” as the type. The samples were obtained from mouse pancreatic tissues, and the sequencing platform file was GPL10787. Sample grouping information is illustrated in Additional file 1: Table S1. Differential analysis of the GSE121038 microarray was performed using the R language “limma” package, with |logFC|> 1, *p* value < 0.05 serving as the threshold to screen DEGs in pancreatitis samples. Additionally, genes related to AP were downloaded from the GeneCards and CDT databases. Intersection analysis was subsequently conducted on the top 300 DEGs in the GSE121038 dataset and the AP-related genes using the jvenn tool. In addition, the binding sequence of transcription factor Rbpjl and the promoter region of Arid5a gene was predicted using the JASPAR website. Lastly, the interaction network of Arid5a and IL-6 was obtained from the STRING website.

### Cell culture and transfection

Mouse pancreatic acinar cell line (MPC-83, CL-0518), procured from Procell (Wuhan, Hubei, China), was cultured in Dulbecco’s modified Eagle’s medium (DMEM) containing 10% fetal bovine serum (FBS) (HyClone Company, Logan, UT), supplemented with 100 U/mL penicillin and 100 U/mL streptomycin (Gibco Company, Grand Island, NY) in a 5% CO_2_ incubator at 37 °C. Upon reaching 80% confluence, the cells were passaged and collected for follow-up experiments.

BLOCK-iT™ RNAi Designer was adopted to design Rbpjl and Arid5a short hairpin RNA (shRNA) sequences. The obtained shRNAs were subsequently cloned into pLKO.1-puro lentivirus plasmids. Mouse Rbpjl and Arid5a full-length sequences were cloned into pCDH-EF1-MCS-copGFP lentivirus plasmids. MPC-83 cells were stimulated with LPS (2 µg/mL, L8880, Beijing Solarbio Science & Technology Co., Ltd., Beijing, China) to mimic the microenvironment of pancreatitis. The specific action time was detailed in the legend of relevant results. In the LPS treatment groups, the cells were transfected with plasmids of oe-NC, oe-Rbpjl, sh-NC, sh-Rbpjl (5′-CCATCCAAACCAGAGTCTGAT-3′), sh-Arid5a (5′-GCCTGAGTATTCAGATGACAA-3′), oe-Rbpjl + oe-NC, oe-Rbpjl + oe-Arid5a, oe-Arid5a + DMSO, and oe-Arid5a + JSI-124 (JSI-124: 1 µM for 24 h, Bio-Techne), alone or in combination. Simultaneously, in control groups, the cells were transfected with plasmids of oe-NC, oe-Rbpjl, sh-NC, sh-Rbpjl, oe-Rbpjl + oe-NC, sh-Arid5a, oe-Rbpjl + oe-Arid5a, oe-Arid5a + DMSO and oe-Arid5a + JSI-124, alone or in combination. Thereafter, the HEK-293 T cells (CL-0005, Procell) were seeded in a 6-well plate, and 4 µg of over-expression or silencing plasmids were added with 8 µL Lipofectamine 3000 (L3000015, Thermo Fisher Scientific Inc., Waltham, MA) for transient transfection. Lentivirus (virus titer of 2 × 10^8^ TU/mL) was collected, isolated, and purified. Afterwards, the purified lentivirus was utilized to transduce MPC-83 cells, which were collected after 72 h.

### RT-qPCR

Tissue or cell samples were lysed with the TRIzol reagent (16,096,020, Thermo Fisher Scientific) for 10–30 min at room temperature. Total RNA content was extracted with the addition of phenol/chloroform, and RNase-Free DNase I was used to digest and extract RNA. The concentration and purity of the extracted RNA (260/280 = 1.8—2.0) were detected with a nucleic acid quantitative analyzer (ND-1000, NanoDrop Technologies Inc.). Next, 400 ng total RNA was reverse-transcribed into cDNA with PrimeScript RT Reagent Kit (D7168l, Beyotime, Shanghai, China). RT-qPCR was subsequently performed using SYBR® Premix Ex Taq™ II kits (RR820A, Takara). PCR primers were synthesized by Riobio Biotechnology Co., Ltd (Guangzhou, China). The specific sequences are illustrated in Additional file 4: Table S4. Glyceraldehyde-3-phosphate dehydrogenase (GAPDH) was adopted as internal reference control of mRNAs. The 2^−ΔΔCT^ method was utilized to express the relative expression of the gene.

### Western blot assay

Total protein was extracted from tissues or cells using radioimmunoprecipitation assay (RIPA) buffer (R0010, Solarbio) with 1 mM PMSF protease inhibitor. BestBio Beibo Nuclear Protein Extraction Kit (BB-3102–1, Invent Biotechnologies Inc., Eden Prairie, MN) was used to extract nuclear protein. Briefly, appropriate amounts of cytoplasmic extraction buffer were added to the cells or tissues, and the homogenate was shaken and centrifuged at 16,000 rpm and 4 °C for 5 min. Next, the supernatant (cytoplasmic fraction) was transferred to a new pre-cooled 1.5 ml centrifuge tube. The pellet was resuspended in 0.5 mL of pre-cooled PBS, and then centrifuged at 10,000 rpm for 3–5 min. The collected pellet was supplemented with an appropriate amount of nucleus extraction buffer, and later incubated on ice for 1 min. Afterwards, the nuclear extract was centrifuged at 14,000–16,000 rpm for 30 s, and the nuclear protein was stored at − 80 °C.

The protein concentration was determined with a bicinchoninic acid (BCA) protein assay kit (G3522, Jiebeisi Biotechnology, Guangzhou, China). Next, 40 µg protein was extracted from each sample and separated by sodium dodecyl sulfate polyacrylamide gel electrophoresis. The obtained proteins were transferred onto a polyvinylidene fluoride (PVDF) membrane (Millipore Corporation, Billeria, MA), which was sealed with TBST containing 5% bovine serum albumin (BSA) at room temperature. The membrane was subsequently incubated with diluted primary antibodies against PAP-I (0.1 µg/mL, PA5-47,700, Thermo Fisher Scientific), Rbpjl (dilution ratio of 1:1000, ab25949, Abcam Inc., Cambridge, UK), Arid5a (dilution ratio of 1:2000, ab81149, Abcam), phosphorylated (p)-STAT3 (dilution ratio of 1:1000, ab76315, Abcam), STAT3 (dilution ratio of 1:1000, _#9139, Cell Signaling Technologies [CST], Beverly, MA), tumor necrosis factor-α (TNF-α) (dilution ratio of 1:1000, #3707, CST), IL-6 (dilution ratio of 1:1000, # 12,153, CST), IL-1β (dilution ratio of 1:1000, #12,153, CST), histone H3 (dilution ratio of 1:1000, # 4499, CST) and GAPDH (dilution ratio of 1:2000, TA-08, Zhongshan Gold-bridge, Beijing, China) overnight at 4 °C. The following day, secondary goat anti-rabbit against IgG antibody (dilution ratio of 1:2000, Zhongshan Gold-bridge) or goat anti-mouse against IgG (dilution ratio of 1:2000, Zhongshan Gold-bridge) was added to the membrane for 1 h incubation at room temperature. Enhanced chemiluminescence reagent (Beyotime) was employed to visualize images using the Bio-Rad Gel Doc XR gel imager (Bio-Rad Laboratories, Hercules, CA). Image J software was adopted to quantify the gray value of the protein bands. The semi-quantitative expression of protein was expressed by the ratio of gray value of the target protein to that of internal reference protein. GAPDH was adopted as the internal reference of total protein, and histone 3 as the internal reference of nuclear protein.

### EMSA

Initially, 1 µg of purified Rbpj1 protein was incubated with 1 μL DNA probe (0.08 μM) in the Arid5a promoter binding region (WT sequence: forward: 5′-CCACCAAGCTCACTGGTGTCTGCTCCAGGCTGAGC-3′; reverse: 5′-GCTCAGCCTGGAGCAGACACCAGTGAGCTTGGTGG-3′; MUT sequence: forward: 5′-CCACCAAGTTTTTTTTTTTTTTCTCCAGGCTGAGC-3′; reverse: 5′-GCTCAGCCTGGAGAAAAAAAAAAAAAACTTGGTGG-3′) in a binding buffer supplemented with 25 mM HEPES buffer (pH 7.6), 50 mM KCl, 0.1 mM EDTA (pH 8.0), 12.5 mM MgCl_2_, 1 mM DTT, 0.5% BSA, and 5% glycerol at room temperature for 30 min. Subsequently, the binding reaction mixture was loaded onto a 7% non-denaturing polyacrylamide gel and run at 80 V for 2 h. Afterwards, the bound DNA was visualized by ethidium bromide staining [37].

### CCK-8 assay

Pancreatic acinar cells were trypsinized and dispersed into a single cell suspension. Next, the cells were seeded into 96-well plates, at a density of 6 × 10^3^ cells per well. Subsequently, 200 μL cell suspension was added to each well, with 6 parallel wells set for each group. At the 24 h, 48 h, 72 h and 96 h time intervals, 10 μL CCK-8 solution (96,992, Sigma-Aldrich Chemical Company, St Louis, MO) was added to each well. After a 2 h period of culture, the optical density (OD) values at 450 nm were detected with a microplate reader (NYW-96 M, Beijing N.Y.W. Instrument Co., Ltd., Beijing, China).

### Flow cytometry

Early and late apoptotic MPC-83 cells were detected by flow cytometry. Briefly, MPC-83 cells (at a density of 1 × 10^4^/well) were trypsinized, rinsed with PBS and resuspended in a binding buffer. Subsequently, the cells were stained with 5 µL Annexin V FITC and PI (BD Biosciences) for 30 min in conditions void of light at 37 °C. The cells were subsequently detected on a flow cytometer (FACScan™; BD Biosciences), and the results were analyzed with the FlowJo software (version 10.6.2; BD Biosciences) to count the proportion of apoptotic cells.

### ROS production assay by dihydroethidium (DHE) fluorescence staining

DHE-ROS reactive oxygen detection kits (BB-47051, Shanghai Best Biotechnology Co., Ltd., Shanghai, China) were adopted to detect ROS accumulation in pancreatic acinar cells. Briefly, DHE solution was diluted with PBS buffer at a ratio of 1:200, added to pancreatic acinar cells, incubated at 37 °C in conditions void of light for 30 min, and then rinsed thrice with PBS. Finally, the cells were visualized under an inverted fluorescence microscope (BX63, Olympus Optical Co., Ltd., Tokyo, Japan). Following excitation by blue light, ROS-positive cells presented with red staining in the whole nuclear area; meanwhile, following excitation with ultraviolet light, the unoxidized dihydroethidine in the cytoplasm presented with blue fluorescence.

### ChIP assay

Cultured pancreatic acinar cells were cross-linked with 1% paraformaldehyde at room temperature for 5 min, and then added with glycine at a final concentration of 127 mM to terminate the cross-linking. Next, the cells were lysed with cell lysis buffer (50 mM Tris HCl, pH = 8.1, 1% sodium dodecyl sulfate, 10 mM EDTA) containing protease inhibitor on ice for 30 min, and then subjected to ultrasonification to produce 200—1000 bp chromatin fragments.

Afterwards, ChIP Assay Kits (#9002, CST) were utilized. Protein A/G agarose (SC-2003, Santa Cruz Biotechnology, Inc, Santa Cruz, CA) and ChIP antibody to Rbpjl (ab25949, Abcam) or IgG (#14,705, CST) were added to 50 μg DNA and incubated overnight at 4 °C. Normal IgG antibody was adopted as NC. The DNA complex was washed successively with low-salt buffer and high-salt buffer. DNA was subsequently extracted and purified with phenol/chloroform. Specific ChIP-RT-qPCR primers (forward: 5′-TTGGAAAGGATGGAACCGGC-3′; reverse: 5′-CTTTGCAGCTAGGGGCTGAT-3′) were designed for the Arid5a promoter.

### Dual luciferase reporter assay

Arid5a promoter region with WT and MUT Rbpjl binding site was constructed into the pGL3-Basic vector (Promega Corp., Madison, Wisconsin) to obtain the recombinant vector of WT-Arid5a promoter (5′-CAGACACCAGTGAG-3′) and MUT-Arid5a promoter (5′-CAGGACTTGACGAG-3′). Subsequently, HEK-293 T cells (CL-0005, Procell) were seeded in 24-well plates, at a density of 3 × 10^4^ cells/well. WT-Arid5a promoter and MUT-Arid5a promoter were then co-transfected with oe-NC and oe-Rbpjl into HEK-293 T cells. At a 48-h period of transfection, the cells were collected and lysed, after which the luciferase activity was detected using the luciferase detection kit (K801-200, BioVision, Milpitas, CA) on a Glomax20/20 Luminometer (Promega). The relative luciferase activity was expressed as the ratio of the firefly luciferase activity to the Renilla luciferase activity. The relative luciferase activity of different treatment groups was normalized to the ratio of 1 of the control group.

### TUNEL staining

TUNEL cell apoptosis detection kits (C1098, Beyotime) was adopted for this experiment. Following pre-processing, the cells were treated with 20 µg/mL of proteinase K without DNase at 37 °C, incubated with 50 µL of TUNEL detection solution at 37 °C for 60 min, and then added with DAB (DA1010, Solarbio) as a substrate. Apoptotic cells were subsequently observed under a microscope. Under a light microscope, the nuclei of the apoptotic cells appeared as brown spots, which were manually counted and quantitatively analyzed in a blind manner.

### Detection of MDA and GSH

GSH and MDA production was determined with GSH (r-glutamyl cysteingl + glycine, #S0056, Beyotime) and MDA kits (#S0131, Beyotime), in accordance with the provided instructions. Briefly, pancreatic tissues (100 mg) were lysed with cell lysis buffer (# p0013, Beyotime) on ice for 30 min, followed by 10-min centrifugation at 12,000*g* and 4 °C, with the supernatant harvested. Next, BCA protein concentration determination kit (#2020LES76, Yeasen Biotechnology, Shanghai, China) was utilized to detect the total protein concentration. Afterwards, GSH activity was detected at 340 nm and MDA activity at 520 nm.

### ELISA

Mouse serum or supernatant of pancreatic acinar cells was collected. ELISA kits for proinflammatory factors TNF-α (ab181421), IL-1β (ab214025) and IL-6 (ab46029) were purchased from Abcam. Briefly, 96-well plates were placed in the microplate reader (Thermo Fisher Scientific) to analyze the OD values at 562 nm. A standard curve was subsequently drawn with standard protein concentration as the X axis and OD value as the Y axis.

### Establishment of AP mouse models

Six-week-old C57/B6 male mice were purchased from Beijing Vital River Biological (Beijing, China) and raised in a specific-pathogen-free animal laboratory. The mice were allowed to acclimatize for two weeks, and then randomly assigned into the following 6 groups (with 8 mice in each group): the sham group, the AP group, the oe-NC group (AP mice injected with lentivirus carrying oe-NC), the oe-Rbpjl group (AP mice injected with lentivirus carrying oe-Rbpjl), the oe-Rbpjl + oe-NC group (AP mice injected with lentivirus carrying oe-Rbpjl + oe-NC) and the oe-Rbpjl + oe-Arid5a (AP mice injected with lentivirus carrying oe-Rbpjl + oe-Arid5a). AP was induced with an intraperitoneal injection of supramaximal cerulein (Sigma-Aldrich) at a dose of 100 mg/kg, for a total of ten times with a 1-h interval between injections. Sham-operated mice were subjected to treatment with PBS. To achieve effective interference, 10 μL of lentivirus (final titer of approximately 1 × 10^8^ TU/mL) was injected via tail vein of mice 1 week prior to AP establishment [38]. Lentiviruses carrying oe-Rbpjl and oe-Arid5a were procured from Genechem (Shanghai, China). The titer of lentivirus was above 1 × 10^9^ TU/mL. Each mouse was injected with 300 µL of corresponding lentivirus for a duration of 14 days. Pancreatic tissues were collected and weighed. The organ coefficient was calculated using the following formula: organ coefficient = organ weight (mg)/body weight (g).

### HE staining

Paraffin-embedded tissue sections were stained with hematoxylin (C0007, Baoman Biotechnology Co., Ltd., Shanghai, China) at room temperature for 10 min, hydrolyzed with 1% hydrochloric acid alcohol, and then reverted to blue in 0.6% ammonia. Next, the tissue sections were counterstained with eosin at room temperature. The morphological and structural changes of pancreatic tissues were subsequently observed under an optical microscope (XSP-36, Boshida Optical Instruments Co., Ltd., Shenzhen, China). Pathological scoring was evaluated, and the parameters included edema, fat necrosis, hemorrhage, inflammatory cell infiltration, and acinar necrosis. The severity of pancreatic injury and the corresponding score were recorded as follows: 0 point indicates no injury; 1 point indicates mild injury; 2 points indicate moderate injury; 3 points indicates severe injury; 4 points indicates very severe injury [39].

### Immunohistochemical staining

Pancreatic tissues were sectioned at the thickness of 5 μm, dehydrated, treated with 3% hydrogen peroxide, and then subjected to peroxidase blocking with normal goat serum. Immunohistochemical staining was subsequently performed by incubating the tissue sections with primary antibody to Rbpjl (dilution ratio of 1:100, 14,613–1-AP, Proteintech) overnight at 4 °C. The following day, the tissues sections were incubated with biotin-labeled goat anti-rabbit secondary antibody (dilution ratio of 1:200, BA1003, Boster Biological Technology Co. Ltd., Wuhan, Hubei, China) for 20 min at 37 °C. Afterwards, the sections were exposed to DAB substrate, followed by hematoxylin counterstaining and standard dehydration treatment. Staining images were obtained using a microscope. Finally, the ImageJ software was adopted for statistical analysis of positive staining. Five different fields of view were randomly selected to count the number of positive cells, and the total number of cells counted was more than 100. The proportion of positive cells = (number of positive cells/total cells) × 100% [40].

### Statistical analysis

Statistical analyses were performed using the SPSS 21.0 statistical software (IBM Corp. Armonk, NY). Measurement data, obtained from three independent experiments, were expressed as mean ± standard deviation. Data between two groups were compared using unpaired *t* test, and those among multiple groups were compared with one-way analysis of variance (ANOVA), combined with Tukey’s post-hoc tests. Data at different time points among multiple groups were compared by repeated measures ANOVA, followed by Bonferroni post-hoc tests. A value of *p* < 0.05 was regarded statistically significant.

## Supplementary Information

**Additional file 1: Table S1.** Sample grouping in the GSE121038 microarray dataset

**Additional file 2: Table S2.** Detailed differential analysis results of the 32 genes obtained from the GSE121038 dataset.

**Additional file 3: Table S3.** Expression data of the 32 genes in control and acute pancreatitis samples of the GSE121038 dataset.

**Additional file 4: Table S4.** Primer sequences for RT-qPCR. Arid5a, AT-rich interactive domain-containing protein 5a; GAPDH, glyceraldehyde-3-phosphate dehydrogenase; RT-qPCR, reverse transcription-quantitative polymerase chain reaction; F, forward; R, reverse.

**Additional file 5: Figure S1.** Detection of pancreatic tissue injury in mice and the expression of pro-inflammatory factors in the cell supernatant of pancreatic acinar cells. A, HE staining for the severity of pancreatic tissue injury in sham-operated or AP mice (* *p* < 0.05 *vs.* sham-operated mice). B, The weight ratio of pancreas to total body of sham-operated or AP mice (* *p* < 0.05 *vs.* sham-operated mice). C, Western blot assay detection of PAP-I protein in control or LPS-induced pancreatic acinar cells (for 24 h). * *p* < 0.05 *vs.* control cells. D, The expression of pro-inflammatory factors in the supernatant of control or LPS-induced pancreatic acinar cells (for 24 h) detected by ELISA. * *p* < 0.05 *vs.* control cells. E, The expression of pro-inflammatory factors in the supernatant of pancreatic acinar cells with oe-Rbpjl or sh-Rbpjl (for 72 h) stimulated by LPS (for 24 h) was detected by ELISA. * *p* < 0.05 *vs.* cells treated with LPS + oe-NC. ^#^ *p* < 0.05 *vs.* cells treated with LPS + sh-NC. The mouse experiments were performed 14 days after AP modeling, with n = 8 for mice in each group. The cell experiment was conducted three times independently.

**Additional file 6: Figure S2.** Representative Western blots. A, Representative Western blots of Figure 3C. B, Representative Western blots of Figure 3I. C, Representative Western blots of Figure 4C. D, Representative Western blots of Figure 4D. E, Representative Western blots of Figure 5B. F, Representative Western blots of Figure 5I. G, Representative Western blots of Figure 5J. H, Representative Western blots of Figure 6A. I, Representative Western blots of Figure 6B. The mouse experiments were performed 14 days after AP modeling, with n = 8 for mice in each group. The cell experiment was conducted three times independently.

**Additional file 7: Figure S3.** Detection of the Rbpjl protein expression and expression of pro-inflammatory factors in pancreatic acinar cells. A, RT-qPCR and Western blot assay were used to detect the protein expression of Rbpjl after pancreatic acinar cells with oe-Rbpjl or sh-Rbpjl (for 72 h) were stimulated by LPS (for 24 h) (* *p* < 0.05 *vs.* cells treated with sh-NC. ^#^ *p* < 0.05 *vs.* cells treated with oe-NC). B, The expression of pro-inflammatory factors in the supernatant of pancreatic acinar cells with oe-Rbpjl or combined with oe-Arid5a (for 72 h) stimulated by LPS (for 24 h) was detected by ELISA. (* *p* < 0.05 *vs.* cells treated with LPS + oe-NC. ^#^ *p* < 0.05 *vs.* cells treated with LPS + oe-Rbpjl + oe-NC) C, The expression of pro-inflammatory factors in the supernatant of pancreatic acinar cells with oe-Arid5a (for 72 h) or combined with JSI-124 (for 24 h) stimulated by LPS (for 24 h) was detected by ELISA. (& *p* < 0.05 *vs.* cells treated with LPS + oe-Arid5a + DMSO) The cell experiment was conducted three times independently.

**Additional file 8: Figure S4.** Rbpjl downregulates Arid5a expression and thus inhibits the IL-6/STAT3 axis to alleviate inflammatory response in control pancreatic acinar cells. A, mRNA expression of Rbpjl, Arid5a and IL-6 in control MPC-83 cells treated with oe-Rbpjl or combined with oe-Arid5a for 72 h determined by RT-qPCR. B, Rbpjl, Arid5a and STAT3 protein expression in MPC-83 cells treated with oe-Rbpjl or combined with oe-Arid5a for 72 h determined by Western blot assay. C, CCK-8 assay was used to detect control MPC-83 cell viability following treatment with oe-Rbpjl or combined with oe-Arid5a for 72 h. D, CCK-8 assay was used to detect control MPC-83 cell viability following treatment with oe-Arid5a or combined with JSI-124 for 72 h. E, Apoptosis of control MPC-83 cells following treatment with oe-Rbpjl or combined with oe-Arid5a for 72 h detected by flow cytometry. F, Apoptosis of control MPC-83 cells following treatment with oe-Arid5a or combined with JSI-124 for 72 h detected by flow cytometry. G, Ultraviolet spectrophotometers were used to detect GSH and MDA production in control MPC-83 cells following treatment with oe-Rbpjl or combined with oe-Arid5a for 72 h. H, Ultraviolet spectrophotometers were used to detect GSH and MDA production in control MPC-83 cells following treatment with oe-Arid5a or combined with JSI-124 for 72 h. I-J, TNF-α, IL-1β and IL-6 expression in control MPC-83 cells following treatment with oe-Arid5a or combined with JSI-124 for 72 h. * *p* < 0.05 *vs.* control MPC-83 cells treated with oe-NC. ^#^ *p* < 0.05 *vs.* control MPC-83 cells treated with oe-Rbpjl + oe-NC. The cell experiment was conducted three times independently.

## Data Availability

The authors confirm that the data supporting the findings of this study are available within the article.

## Abbreviations

AP: Acute pancreatitis
LPS: Lipopolysaccharide
ROS: Reactive oxygen species
Rbpjl: Recombination signal binding protein for immunoglobulin kappa J region like
PTF1: Pancreas transcription factor 1
Arid5a: AT-rich interactive domain-containing protein 5a
IL-6: Interleukin 6
STAT3: Signal transducers and activators of transcription 3
LPS: Lipopolysaccharide
DEGs: Differentially expressed genes
HE: Hematoxylin and eosin
TUNEL: Terminal deoxynucleotidyl transferase-mediated dUTP-biotin nick end labeling
ELISA: Enzyme-linked immunosorbent assay
PAP-I: Pancreatitis-associated protein-I
GSH: Glutathione
MDA: Malondialdehyde
ROS: Reactive oxygen species
RT-qPCR: Reverse transcription-quantitative polymerase chain reaction
ChIP: Chromatin immunoprecipitation
EMSA: Electrophoresis mobility shift assay
CCK-8: Cell counting kit-8
FBS: Fetal bovine serum
shRNA: Short hairpin RNA
GAPDH: Glyceraldehyde-3-phosphate dehydrogenase
RIPA: Radioimmunoprecipitation assay
BCA: Bicinchoninic acid
PVDF: Polyvinylidene fluoride
BSA: Bovine serum albumin
HEPES: N-2-hydroxyethyl-piperazine-N'-2-ethanesulfonic acid
EDTA: Ethylenediamine tetraacetic acid
DTT: Dithiothreitol
OD: Optical density
DHE: Dihydroethidium
ANOVA: Analysis of variance

## References

[1] Garg PK, Singh VP (2019). Organ failure due to systemic injury in acute pancreatitis. Gastroenterology.

[2] Boxhoorn L, Voermans RP, Bouwense SA, Bruno MJ, Verdonk RC, Boermeester MA (2020). Acute pancreatitis. Lancet.

[3] Dios ID (2010). Inflammatory role of the acinar cells during acute pancreatitis. World J Gastrointest Pharmacol Ther.

[4] Lee PJ, Papachristou GI (2019). New insights into acute pancreatitis. Nat Rev Gastroenterol Hepatol.

[5] Saluja A, Dudeja V, Dawra R, Sah RP (2019). Early intra-acinar events in pathogenesis of pancreatitis. Gastroenterology.

[6] Nair AK, Sutherland JR, Traurig M, Piaggi P, Chen P, Kobes S (2018). Functional and association analysis of an Amerindian-derived population-specific p.(Thr280Met) variant in RBPJL, a component of the PTF1 complex. Eur J Hum Genet.

[7] Danielsson A, Ponten F, Fagerberg L, Hallstrom BM, Schwenk JM, Uhlen M (2014). The human pancreas proteome defined by transcriptomics and antibody-based profiling. PLoS ONE.

[8] Masui T, Swift GH, Deering T, Shen C, Coats WS, Long Q (2010). Replacement of Rbpj with Rbpjl in the PTF1 complex controls the final maturation of pancreatic acinar cells. Gastroenterology.

[9] Higa M, Oka M, Fujihara Y, Masuda K, Yoneda Y, Kishimoto T (2018). Regulation of inflammatory responses by dynamic subcellular localization of RNA-binding protein Arid5a. Proc Natl Acad Sci U S A.

[10] Nyati KK, Zaman MM, Sharma P, Kishimoto T (2020). Arid5a, an RNA-Binding Protein in Immune Regulation: RNA Stability, Inflammation, and Autoimmunity. Trends Immunol.

[11] Zaman MM, Masuda K, Nyati KK, Dubey PK, Ripley B, Wang K (2016). Arid5a exacerbates IFN-gamma-mediated septic shock by stabilizing T-bet mRNA. Proc Natl Acad Sci U S A.

[12] Tanaka T, Narazaki M, Kishimoto T (2018). Interleukin (IL-6) Immunotherapy. Cold Spring Harb Perspect Biol.

[13] Hu YS, Han X, Liu XH (2019). STAT3: A Potential Drug Target for Tumor and Inflammation. Curr Top Med Chem.

[14] Li J, Pan X, Yang J, Jia L, Wu C, Liu H (2020). Enteral virus depletion modulates experimental acute pancreatitis via toll-like receptor 9 signaling. Biochem Pharmacol.

[15] Miao B, Qi WJ, Zhang SW, Wang H, Wang C, Hu L (2019). miR-148a suppresses autophagy by down-regulation of IL-6/STAT3 signaling in cerulein-induced acute pancreatitis. Pancreatology.

[16] Prevot PP, Augereau C, Simion A, Van den Steen G, Dauguet N, Lemaigre FP (2013). Let-7b and miR-495 stimulate differentiation and prevent metaplasia of pancreatic acinar cells by repressing HNF6. Gastroenterology.

[17] Hale MA, Swift GH, Hoang CQ, Deering TG, Masui T, Lee YK (2014). The nuclear hormone receptor family member NR5A2 controls aspects of multipotent progenitor cell formation and acinar differentiation during pancreatic organogenesis. Development.

[18] Dusetti NJ, Ortiz EM, Mallo GV, Dagorn JC, Iovanna JL (1995). Pancreatitis-associated protein I (PAP I), an acute phase protein induced by cytokines. Identification of two functional interleukin-6 response elements in the rat PAP I promoter region. J Biol Chem.

[19] Nyati KK, Agarwal RG, Sharma P, Kishimoto T (2019). Arid5a Regulation and the Roles of Arid5a in the Inflammatory Response and Disease. Front Immunol.

[20] Masui T, Long Q, Beres TM, Magnuson MA, MacDonald RJ (2007). Early pancreatic development requires the vertebrate Suppressor of Hairless (RBPJ) in the PTF1 bHLH complex. Genes Dev.

[21] Molero X, Vaquero EC, Flandez M, Gonzalez AM, Ortiz MA, Cibrian-Uhalte E (2012). Gene expression dynamics after murine pancreatitis unveils novel roles for Hnf1alpha in acinar cell homeostasis. Gut.

[22] Krah NM, De La OJ, Swift GH, Hoang CQ, Willet SG, Chen Pan F (2015). The acinar differentiation determinant PTF1A inhibits initiation of pancreatic ductal adenocarcinoma. Elife.

[23] Dubey PK, Masuda K, Nyati KK, Uz Zaman MM, Chalise JP, Millrine D (2017). Arid5a-deficient mice are highly resistant to bleomycin-induced lung injury. Int Immunol.

[24] Masuda K, Ripley B, Nishimura R, Mino T, Takeuchi O, Shioi G (2013). Arid5a controls IL-6 mRNA stability, which contributes to elevation of IL-6 level in vivo. Proc Natl Acad Sci U S A.

[25] Nyati KK, Masuda K, Zaman MM, Dubey PK, Millrine D, Chalise JP (2017). TLR4-induced NF-kappaB and MAPK signaling regulate the IL-6 mRNA stabilizing protein Arid5a. Nucleic Acids Res.

[26] Masuda K, Kishimoto T (2018). A Potential Therapeutic Target RNA-binding Protein, Arid5a for the treatment of inflammatory disease associated with aberrant cytokine expression. Curr Pharm Des.

[27] Tanaka S, Imaeda A, Matsumoto K, Maeda M, Obana M, Fujio Y (2020). beta2-adrenergic stimulation induces interleukin-6 by increasing Arid5a, a stabilizer of mRNA, through cAMP/PKA/CREB pathway in cardiac fibroblasts. Pharmacol Res Perspect.

[28] Masuda K, Ripley B, Nyati KK, Dubey PK, Zaman MM, Hanieh H (2016). Arid5a regulates naive CD4+ T cell fate through selective stabilization of Stat3 mRNA. J Exp Med.

[29] Dong J, Chen X, Song Y, Fei X (2019). Chaiqin Chengqi decoction inhibits inflammatory mediators and attenuates acute pancreatitis through deactivation of janus kinase/signal transducer and activator of transcription signaling pathway. J Tradit Chin Med.

[30] Wang Q, Bai L, Luo S, Wang T, Yang F, Xia J (2020). TMEM16A Ca(2+)-activated Cl(-) channel inhibition ameliorates acute pancreatitis via the IP3R/Ca(2+)/NFkappaB/IL-6 signaling pathway. J Adv Res.

[31] Piao X, Liu B, Sui X, Li S, Niu W, Zhang Q (2020). Picroside II Improves Severe Acute Pancreatitis-Induced Intestinal Barrier Injury by Inactivating Oxidative and Inflammatory TLR4-Dependent PI3K/AKT/NF-kappaB Signaling and Improving Gut Microbiota. Oxid Med Cell Longev.

[32] Khavinson V, Durnova AO, Polyakova VO, Tolibova GH, Linkova NS, Kvetnoy IM (2013). Effects of pancragen on the differentiation of pancreatic cells during their ageing. Bull Exp Biol Med.

[33] Sugiyama T, Benitez CM, Ghodasara A, Liu L, McLean GW, Lee J (2013). Reconstituting pancreas development from purified progenitor cells reveals genes essential for islet differentiation. Proc Natl Acad Sci U S A.

[34] Hua J, He ZG, Qian DH, Lin SP, Gong J, Meng HB (2014). Angiopoietin-1 gene-modified human mesenchymal stem cells promote angiogenesis and reduce acute pancreatitis in rats. Int J Clin Exp Pathol.

[35] Morita S, Hara A, Kojima I, Horii T, Kimura M, Kitamura T (2009). Dicer is required for maintaining adult pancreas. PLoS ONE.

[36] Manohar M, Verma AK, Venkateshaiah SU, Sanders NL, Mishra A (2017). Pathogenic mechanisms of pancreatitis. World J Gastrointest Pharmacol Ther.

[37] Tan LM, Liu R, Gu BW, Zhang CJ, Luo J, Guo J (2020). Dual Recognition of H3K4me3 and DNA by the ISWI Component ARID5 Regulates the Floral Transition in Arabidopsis. Plant Cell.

[38] Ku MW, Authie P, Nevo F, Souque P, Bourgine M, Romano M (2021). Lentiviral vector induces high-quality memory T cells via dendritic cells transduction. Commun Biol.

[39] Wen Y, Liu R, Lin N, Luo H, Tang J, Huang Q (2019). NADPH oxidase hyperactivity contributes to cardiac dysfunction and apoptosis in rats with severe experimental pancreatitis through ROS-Mediated MAPK Signaling Pathway. Oxid Med Cell Longev.

[40] Bian SB, Yang Y, Liang WQ, Zhang KC, Chen L, Zhang ZT (2021). Leukemia inhibitory factor promotes gastric cancer cell proliferation, migration, and invasion via the LIFR-Hippo-YAP pathway. Ann N Y Acad Sci.

