# Supplementary material for: Blockade of the Arid5a/IL-6/STAT3 axis underlies the anti-inflammatory effect of Rbpjl in acute pancreatitis
Source: Cell Biosci. 2022 Jun 20;12:95. doi: 10.1186/s13578-022-00819-1 (PMC9208186; doi:10.1186/s13578-022-00819-1)
Supplement: Supplementary file 1 — Additional file 1: Table S1. Sample grouping in the GSE121038 microarray dataset [file 13578_2022_819_MOESM1_ESM.docx]

**Supplementary Table 1** Sample grouping in the GSE121038 microarray dataset

| GEO accession | Control | Acute pancreatitis (AP) |
| --- | --- | --- |
| GSE121038 | GSM3424904  GSM3424905  GSM3424906  GSM3424907 | GSM3424908  GSM3424909  GSM3424910  GSM3424911 |
