# Supplementary material for: Blockade of the Arid5a/IL-6/STAT3 axis underlies the anti-inflammatory effect of Rbpjl in acute pancreatitis
Source: Cell Biosci. 2022 Jun 20;12:95. doi: 10.1186/s13578-022-00819-1 (PMC9208186; doi:10.1186/s13578-022-00819-1)
Supplement: Supplementary file 2 — Additional file 2: Table S2. Detailed differential analysis results of the 32 genes obtained from the GSE121038 dataset. [file 13578_2022_819_MOESM2_ESM.docx]

**Supplementary Table 2** Detailed differential analysis results of the 32 genes obtained from the GSE121038 dataset

| Gene | logFC | AveExpr | t | *p* value | adj.P.Val | B |
| --- | --- | --- | --- | --- | --- | --- |
| JUN | 4.109194 | 11.69677 | 10.71135 | 2.1E-06 | 0.000266 | 5.5516 |
| CCL2 | 3.0677 | 7.473844 | 11.28499 | 1.35E-06 | 0.000211 | 5.995161 |
| RELA | 2.240248 | 13.24172 | 16.93407 | 4.15E-08 | 2.51E-05 | 9.371253 |
| CXCL1 | 2.641744 | 5.080505 | 12.07352 | 7.64E-07 | 0.000146 | 6.568291 |
| CDKN1A | 1.935022 | 7.136935 | 11.42286 | 1.22E-06 | 0.000198 | 6.09833 |
| ATF4 | 2.162541 | 12.93774 | 11.73748 | 9.7E-07 | 0.00017 | 6.328998 |
| GADD45A | 5.562808 | 8.47565 | 20.0759 | 9.37E-09 | 1.17E-05 | 10.69935 |
| CEBPB | 3.089399 | 11.83745 | 16.42642 | 5.41E-08 | 2.63E-05 | 9.12647 |
| CDH1 | 2.382025 | 10.39449 | 11.89953 | 8.64E-07 | 0.00016 | 6.445286 |
| GDF15 | 4.160818 | 9.393671 | 16.48144 | 5.26E-08 | 2.63E-05 | 9.153455 |
| THBS1 | 3.442872 | 8.536611 | 12.8095 | 4.62E-07 | 0.000108 | 7.068334 |
| FLNB | 1.815934 | 9.793505 | 11.06848 | 1.59E-06 | 0.000221 | 5.830496 |
| SAT1 | 2.051753 | 12.37655 | 9.858747 | 4.18E-06 | 0.000417 | 4.846546 |
| MCL1 | 1.283805 | 11.64903 | 8.90653 | 9.61E-06 | 0.000724 | 3.986837 |
| FOXA2 | -2.64953 | 8.767908 | -9.24856 | 7.07E-06 | 0.000573 | 4.30503 |
| HBEGF | 5.488134 | 8.9745 | 23.01657 | 2.81E-09 | 6.24E-06 | 11.705 |
| HSPB1 | 2.978052 | 13.85511 | 10.15903 | 3.26E-06 | 0.000351 | 5.101434 |
| CHKA | 2.730057 | 9.805697 | 12.2186 | 6.9E-07 | 0.000139 | 6.669425 |
| MTHFR | 1.311708 | 7.85553 | 9.787385 | 4.44E-06 | 0.000428 | 4.784875 |
| ACTB | 2.836145 | 16.29788 | 9.317311 | 6.65E-06 | 0.000567 | 4.36769 |
| CTNNA1 | 1.155899 | 12.07925 | 9.247945 | 7.07E-06 | 0.000573 | 4.304472 |
| KRT8 | 4.405147 | 14.35088 | 23.66713 | 2.2E-09 | 6.24E-06 | 11.90222 |
| KRT18 | 2.213226 | 16.29088 | 13.34583 | 3.25E-07 | 8.92E-05 | 7.413278 |
| RASSF1 | 1.880614 | 11.08491 | 10.09417 | 3.44E-06 | 0.000363 | 5.047004 |
| PRDM16 | -2.63778 | 6.284827 | -10.5807 | 2.32E-06 | 0.00028 | 5.447184 |
| ANGPT1 | -2.15903 | 6.724157 | -8.84126 | 1.02E-05 | 0.000751 | 3.924858 |
| CLDN4 | 3.229099 | 7.410034 | 11.94991 | 8.34E-07 | 0.000155 | 6.481099 |
| SBDS | 1.693282 | 11.46183 | 11.33578 | 1.3E-06 | 0.000205 | 6.03332 |
| DICER1 | -1.68953 | 7.239272 | -9.68492 | 4.84E-06 | 0.000453 | 4.695576 |
| NEAT1 | 2.899409 | 14.57862 | 12.28981 | 6.57E-07 | 0.000138 | 6.718589 |
| PTF1A | 1.464605 | 13.00221 | 9.950565 | 3.87E-06 | 0.000398 | 4.925268 |
| Rbpjl | -1.87192 | 10.77653 | -9.10972 | 0.000008 | 0.000618 | 4.177184 |
