# Supplementary material for: Blockade of the Arid5a/IL-6/STAT3 axis underlies the anti-inflammatory effect of Rbpjl in acute pancreatitis
Source: Cell Biosci. 2022 Jun 20;12:95. doi: 10.1186/s13578-022-00819-1 (PMC9208186; doi:10.1186/s13578-022-00819-1)
Supplement: Supplementary file 3 — Additional file 3: Table S3. Expression data of the 32 genes in control and acute pancreatitis samples of the GSE121038 dataset. [file 13578_2022_819_MOESM3_ESM.docx]

**Supplementary Table 3** Expression data of the 32 genes in control and acute pancreatitis samples of the GSE121038 dataset

| id | GSM3424904 | GSM3424905 | GSM3424906 | GSM3424907 | GSM3424908 | GSM3424909 | GSM3424910 | GSM3424911 |
| --- | --- | --- | --- | --- | --- | --- | --- | --- |
| JUN | 9.332331 | 10.04608 | 9.651917 | 9.538354 | 14.33203 | 14.20267 | 12.5083 | 13.96247 |
| CCL2 | 6.26308 | 5.663431 | 5.631297 | 6.202168 | 9.669298 | 8.570316 | 8.684499 | 9.106662 |
| RELA | 12.09351 | 12.06445 | 12.08744 | 12.24096 | 14.3136 | 14.35578 | 14.22356 | 14.55443 |
| CXCL1 | 3.595269 | 3.594523 | 3.704867 | 4.143872 | 6.342082 | 6.622917 | 6.739434 | 5.901075 |
| CDKN1A | 6.16134 | 6.41401 | 6.334619 | 5.767726 | 8.188339 | 8.193471 | 8.010611 | 8.025363 |
| ATF4 | 11.47465 | 12.04315 | 12.16795 | 11.74013 | 14.14662 | 14.17498 | 13.86029 | 13.89416 |
| GADD45A | 5.376388 | 5.617518 | 6.025684 | 5.757394 | 11.52184 | 11.53366 | 10.42696 | 11.54576 |
| CEBPB | 10.23071 | 10.30412 | 10.35915 | 10.27704 | 13.5076 | 13.6483 | 12.85001 | 13.52272 |
| CDH1 | 9.062168 | 9.201882 | 9.454239 | 9.095626 | 11.60781 | 11.85791 | 11.07093 | 11.80536 |
| GDF15 | 7.151035 | 7.45861 | 7.917366 | 6.726036 | 11.55047 | 11.7513 | 11.34031 | 11.25423 |
| THBS1 | 6.326559 | 7.186115 | 6.763848 | 6.984176 | 9.836779 | 10.87005 | 9.961735 | 10.36362 |
| FLNB | 8.821393 | 8.872469 | 9.024593 | 8.823698 | 10.7733 | 10.96382 | 10.32293 | 10.74584 |
| SAT1 | 11.43689 | 11.47465 | 11.23847 | 11.25268 | 12.99086 | 13.58437 | 13.88228 | 13.1522 |
| MCL1 | 10.76199 | 11.03998 | 10.96109 | 11.26546 | 12.27631 | 12.29244 | 12.35403 | 12.24096 |
| FOXA2 | 9.983119 | 10.12064 | 10.11844 | 10.14848 | 7.421296 | 8.262985 | 6.707241 | 7.381053 |
| HBEGF | 5.89118 | 5.88786 | 6.789849 | 6.352843 | 12.02871 | 11.7592 | 11.72093 | 11.36543 |
| HSPB1 | 12.70449 | 12.47872 | 11.93637 | 12.34477 | 14.77355 | 16.12616 | 15.36485 | 15.11199 |
| CHKA | 7.845561 | 8.748272 | 8.757175 | 8.411665 | 11.11332 | 11.17922 | 11.42367 | 10.9667 |
| MTHFR | 7.320854 | 7.254461 | 7.176781 | 7.04661 | 8.640731 | 8.58908 | 8.422825 | 8.3929 |
| ACTB | 15.19554 | 14.57806 | 14.26687 | 15.47878 | 18.12186 | 17.83884 | 17.15212 | 17.751 |
| CTNNA1 | 11.44752 | 11.49131 | 11.54453 | 11.52184 | 12.66481 | 12.79323 | 12.51386 | 12.6569 |
| KRT8 | 12.10652 | 12.29712 | 12.1947 | 11.99488 | 16.5202 | 16.90967 | 16.11325 | 16.67069 |
| KRT18 | 15.34013 | 15.10343 | 15.18154 | 15.11199 | 17.43769 | 17.48105 | 17.01558 | 17.65566 |
| RASSF1 | 10.01188 | 10.25652 | 10.1937 | 10.11632 | 11.57197 | 12.34229 | 11.95602 | 12.23059 |
| PRDM16 | 7.446489 | 7.531041 | 7.823116 | 7.614218 | 4.39996 | 5.32672 | 4.663567 | 5.473509 |
| ANGPT1 | 7.215603 | 7.755675 | 8.328898 | 7.914519 | 5.687168 | 5.80655 | 5.270851 | 5.813991 |
| CLDN4 | 5.855404 | 5.794963 | 5.816577 | 5.714994 | 8.464854 | 9.109389 | 8.70139 | 9.8227 |
| SBDS | 10.6023 | 10.6574 | 10.60989 | 10.59116 | 12.00346 | 12.27004 | 12.54418 | 12.4162 |
| DICER1 | 7.662206 | 8.167052 | 8.315611 | 8.191276 | 6.314433 | 6.43786 | 6.253589 | 6.572148 |
| NEAT1 | 12.60678 | 12.84415 | 13.34009 | 13.72465 | 15.96408 | 16.0643 | 16.00739 | 16.07755 |
| PTF1A | 12.11279 | 12.20315 | 12.42904 | 12.33465 | 13.84182 | 13.63944 | 13.53692 | 13.91988 |
| Rbpjl | 11.25423 | 11.76177 | 11.99658 | 11.83738 | 10.08436 | 9.813847 | 9.478628 | 9.985455 |
