# Supplementary material for: Blockade of the Arid5a/IL-6/STAT3 axis underlies the anti-inflammatory effect of Rbpjl in acute pancreatitis
Source: Cell Biosci. 2022 Jun 20;12:95. doi: 10.1186/s13578-022-00819-1 (PMC9208186; doi:10.1186/s13578-022-00819-1)
Supplement: Supplementary file 4 — Additional file 4: Table S4. Primer sequences for RT-qPCR. Arid5a, AT-rich interactive domain-containing protein 5a; GAPDH, glyceraldehyde-3-phosphate dehydrogenase; RT-qPCR, reverse transcription-quantitative polymerase chain reaction; F, forward; R, reverse. [file 13578_2022_819_MOESM4_ESM.docx]

**Supplementary Table 4** Primer sequences for RT-qPCR

| Gene | Sequence |
| --- | --- |
| Arid5a | F: 5'-CGACAAGCCACTGCCTCCTA-3' |
|  | R: 5'-GCATCTGATTTGGTCTTTCCTG-3' |
| Rbpjl | F: 5'-CCAGGAAGGTTACATCCGCTAC-3' |
|  | R: 5'-CATCAAGGAGGGCACACTGCTT-3' |
| IL-6 | F: 5'-TACCACTTCACAAGTCGGAGGC-3' |
|  | R: 5'-CTGCAAGTGCATCATCGTTGTTC-3' |
| GAPDH | F: 5'-CCCTTAAGAGGGATGCTGCC-3' |
|  | R: 5'-ACTGTGCCGTTGAATTTGCC-3' |

Note: Arid5a, AT-rich interactive domain-containing protein 5a; GAPDH, glyceraldehyde-3-phosphate dehydrogenase; RT-qPCR, reverse transcription-quantitative polymerase chain reaction; F, forward; R, reverse.
